# Parameter Optimization and Development of Mini Infrared Lidar for Atmospheric Three-Dimensional Detection

**DOI:** 10.3390/s23020892

**Published:** 2023-01-12

**Authors:** Zhiqiang Kuang, Dong Liu, Decheng Wu, Zhenzhu Wang, Cheng Li, Qian Deng

**Affiliations:** 1 Key Laboratory of Atmospheric Optics, Anhui Institute of Optics and Fine Mechanics, Hefei Institutes of Physical Science, Chinese Academy of Sciences, Hefei 230031, China; 2 Science Island Branch of Graduate School, University of Science and Technology of China, Hefei 230026, China; 3 Advanced Laser Technology Laboratory of Anhui Province, Hefei 230037, China

**Keywords:** lidar, sensor, parameter optimization, atmospheric detection, infrared wavelength

## Abstract

In order to conduct more thorough research on the structural characteristics of the atmosphere and the distribution and transmission of atmospheric pollution, the use of remote sensing technology for multi-dimensional detection of the atmosphere is needed. A light-weight, low-volume, low-cost, easy-to-use and low-maintenance mini Infrared Lidar (mIRLidar) sensor is developed for the first time. The model of lidar is established, and the key optical parameters of the mIRLidar are optimized through simulation, in which wavelength of laser, energy of pulse laser, diameter of telescope, field of view (FOV), and bandwidth of filter are included. The volume and weight of the lidar system are effectively reduced through optimizing the structural design and designing a temperature control system to ensure the stable operation of the core components. The mIRLidar system involved a 1064 nm laser (the pulse laser energy 15 μJ, the repetition frequency 5 kHz), a 100 mm aperture telescope (the FOV 1.5 mrad), a 0.5 nm bandwidth of filter and an APD, where the lidar has a volume of 200 mm × 200 mm × 420 mm and weighs about 13.5 kg. It is shown that the lidar can effectively detect three-dimensional distribution and transmission of aerosol and atmospheric pollution within a 5 km detection range, from Horizontal, scanning and navigational atmospheric measurements. It has great potential in the field of meteorological research and environmental monitoring.

## 1. Introduction

Climate change and environmental pollution are two of the major development related issues currently affecting China and the rest of the world. Atmospheric pollution not only affects climate change, but also seriously threatens the safety of human life. Lidar is widely used in atmospheric detection and environmental monitoring due to its fine time resolution, high spatial resolution, large detection range and real-time continuous detection capability [1,2,3,4], while in-situ monitoring instruments only measure local concentrations. In 1992, NASA successfully developed micro-pulse lidar (MPL) for the detection of particle and clouds [5,6], which is used in the global aerosol detection network Micro-Pluse Lidar Network (MPLNET) [7]. MPL has subsequently been used extensively in atmospheric aerosol and cloud detection. Some researchers have developed scanning lidar for multi-dimensional atmospheric scanning detection. Gong [8] has developed a 1.5 μm scanning lidar for aerosol detection. Xie [9], Yan [10] and Chiang [11] have developed scanning lidar to realize three-dimensional detection of the atmosphere. Mobile lidar was also developed for tropospheric aerosol detection in different places [12]. Mei [13] has developed an imaging lidar named mini-Scheimpflug lidar, and Shiina [14] has developed a LED mini lidar, using LED for light source; these two lidar be used for short distance detection, with small size and low-power consumption.

Measurements of the lower atmospheric boundary layer in three dimensions in metropolitan areas can offer information on aerosol distribution in locations where a large portion of our population lives and works. Based on Lidar technology, with different detection mode (scanning,) three dimensional detection of the atmosphere can be achieved. Overall, development of a light-weight, low-volume, low-cost, easy-to-use and low-maintenance lidar sensor is important and necessary.

In this paper, we apply simulation to optimize the key parameters of the lidar, optimize the design of the structure and strengthen the environmental adaptability of the lidar, then develop a miniaturized infrared lidar sensor. The effective detection distance can reach up to 5 km in most weather conditions. The lidar can work unattended outdoors with a small size, lightweight, low power consumption. The mIRLidar can be used in meteorological research and environmental monitoring.

The rest of this paper is organized as follows. In Section 2, the model of lidar is established and the key parameters of lidar are optimized. In Section 3, We focus on the design of structure and temperature control, and develop the mIRLidar. Then we test the detection performance of mIRLidar and conduct a series of detection experiments in vertical mode, scanning mode and navigation mode as shown in Section 4. In the last section of the paper, conclusions and outlook are demonstrated.

## 2. Parameter Optimization

The parameter simulation and optimization of the mIRLidar system is based on simulation model established with lidar equation and combined with the atmosphere model, using SNR ratio as an evaluation criterion. The model of lidar represents the relationship between each parameter and performance of lidar. Using the design parameter of mIRLidar and atmospheric model, the detection performance of lidar is simulated in different detection mode. The results of simulation can ensure researcher to select optimal parameters when they begin designing, and help them understand mIRLidar system completely. Optimizing parameters and testing system performance through simulation has been carried out in other lidar systems [15], and a complete theory and process has been established [16].

**Figure 1:**
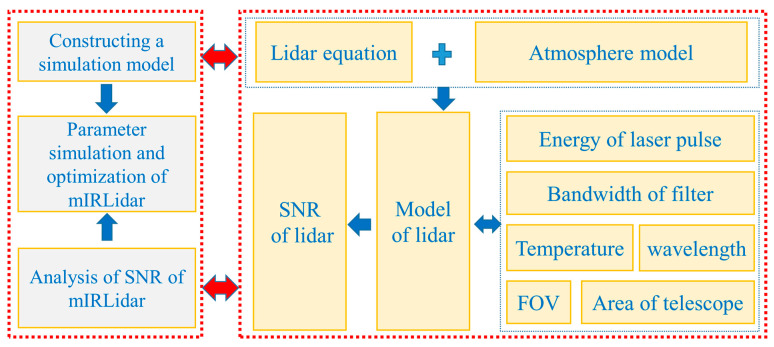
Model of parameter optimization.

Before designing the mIRLidar, we must preliminarily determine the theoretical parameters of each part of the optical components. From Figure 1. on the basis of lidar model and the SNR results, the system design parameters are further determined and optimized. In the simulation of the design parameters of the mIRLidar system, wavelength of laser, energy of pulse laser, diameter of telescope, field of view (FOV) and bandwidth of filter are studied. At the same time, the influence of temperature on SNR is considered. We design the mIRLidar with reference to our previous design experience in mobile lidar [17] and scanning micropulse lidar [18] in terms of device parameter design and device selection.

### 2.1. Model of Lidar

Atmospheric lidar transmits laser pulses into the atmosphere, where aerosols and air molecules interact with them. The backscattering echo signals are collected by the optical telescope unit, and then data acquisition and processing are carried out to invert the parameters of atmospheric aerosols or clouds, such as aerosol extinction coefficient and the height of clouds.

The power of the backscatter signal $P_{R}\left(\lambda ,Z\right)$ received at a distance Z (km) of the transmitting laser wavelength λ (nm) can be expressed as [19]

(1) $$P_{R}\left(\lambda ,Z\right)=2T_{T}E_{T}Z^{-2}cA_{R}\beta \left(\lambda ,Z\right)exp\left[-2\int_{0}^{z}\alpha \left(\lambda ,Z^{\prime }\right)dzZ^{\prime }\right],$$

where $T_{T}$ is the total transmittance of the emission system, $E_{T}$ the energy of a single laser pulse (J), c the speed of light (km s^−1^), $A_{R}$ is the telescope aperture area (km^2^), β(λ, Z) is  the total backscatter coefficient (km^−1^ sr^−1^) and α(λ, Z) is  the total extinction coefficient (km^−1^). The α(λ, Z) and β(λ, Z) are two parameters that characterize the optical properties of the atmosphere as a result of the interaction between the laser and the atmosphere. They are used as input quantities in the simulation of Lidar. Both β(λ,Z)  and α(λ,Z) contain contributions from aerosol particulates and air molecules:

(2) $$\beta \left(\lambda ,Z\right)=\beta_{a}\left(\lambda ,Z\right)+\beta_{m}\left(\lambda ,Z\right),$$

(3) $$\alpha \left(\lambda ,Z\right)=\alpha_{a}\left(\lambda ,Z\right)+\alpha_{m}\left(\lambda ,Z\right),$$

where the subscript a refers to atmospheric aerosols and m refers to molecules. Define aerosol extinction backscatter coefficient ratio $S_{a}\left(\lambda ,\;Z\right)$ (known as aerosol lidar ratio):

(4) $$S_{a}\left(\lambda ,Z\right)=\frac{\alpha_{a}\left(\lambda ,Z\right)}{\beta_{a}\left(\lambda ,Z\right)},$$

In order to solve the lidar equation containing two unknown variables, a linear relationship is assumed to exist between $\alpha_{a}\left(\lambda ,Z\right)$ and $\beta_{a}\left(\lambda ,Z\right)$ [20], so that the lidar equation can be solved to obtain β_a_. The value of $S_{a}\left(\lambda ,Z\right)$ is related to aerosol size scale, refractive index and detection wavelength, and ranges generally between 10 and 90 sr [21]. To simplify the process of simulation analysis, we assume that the atmospheric aerosol is stable and homogeneous, so that $S_{a}$ is set as a constant. $S_{a}=50\;sr$ for 355 nm wavelength, $S_{a}=50\;sr$ for 532 nm wavelength, $S_{a}=40\;sr$ for 1064 nm wavelength [21]. The extinction backscattering ratio of air molecules $S_{m}(\lambda ,Z)$ (known as molecular lidar ratio):

(5) $$S_{m}=\frac{\alpha_{m}\left(\lambda ,Z\right)}{\beta_{m}\left(\lambda ,Z\right)}=8\pi /3,$$

The extinction coefficient of air molecules $\alpha_{m}\left(\lambda ,Z\right)$ can be calculated by Rayleigh scattering theory based on the density of air molecules, which can be obtained from the actual temperature and pressure humidity meteorological sounding data in the atmosphere or using the standard temperature and pressure humidity atmospheric model [22].

Substituting Equations (2)–(5) into Equation (1), we get:

(6) $$P_{R}\left(\lambda ,Z\right)=2T_{T}E_{T}Z^{-2}cA_{R}\left(\frac{\alpha_{m}\left(\lambda ,Z\right)}{S_{m}}+\frac{\alpha_{a}\left(\lambda ,Z\right)}{S_{a}\left(\lambda \right)}\right)exp\left\{-2\int_{0}^{z}\left[\alpha_{m}\left(\lambda ,Z^{\prime }\right)+\;\alpha_{m}\left(\lambda ,Z^{\prime }\right)\right]dZ^{\prime }\right\},$$

A photon detector and photon counting card are used to detect and collect backscatter signal. Photon signal $N_{S}\left(\lambda ,Z\right)$ at distance Z is expressed as follows:

(7) $$N_{S}\left(\lambda ,Z\right)=T_{R}P_{R}\left(\lambda ,Z\right)\Delta t\frac{\eta \lambda }{hc},$$

$T_{R}$ is the optical efficiency of the receiving system, Δt is the sampling time (s), η is the quantum efficiency of the detector, and h is the Planck constant. Sky background noise can be expressed as:

(8) $$N_{B}\left(\lambda \right)=T_{R}P_{B}\left(\lambda \right)\pi {\left(\frac{\theta }{2}\right)}^{2}\Delta \lambda A_{R}\Delta t\frac{\eta \lambda }{hc},$$

$P_{B}\left(\lambda \right)$ is the sky radiation background intensity (W km^−2^ sr^−1^ nm^−1^), θ is the FOV (radians) of telescope, and Δλ is the bandwidth of the interference filter (nm). The signal finally obtained also includes dark count noise from the detector, not only the backscatter signal and sky background noise. The dark count of the detector can be expressed as:

(9) $$N_{D}=C_{D}\Delta t,$$

$C_{D}$ is the detector dark count rate(s^−1^). The total lidar echo signal N(λ,Z) can be expressed as:

(10) $$N\left(\lambda ,Z\right)=N_{S}\left(\lambda ,Z\right)+N_{D}+N_{B}\left(\lambda \right).$$

The SNR of the final echo signal can be evaluated as [5]:

(11) $$SNR\left(\lambda ,Z\right)=\frac{N_{S}\left(\lambda ,Z\right)}{\sqrt{N_{S}\left(\lambda ,Z\right)+2(N_{B}\left(\lambda \right)+N_{D})}}\sqrt{M},$$

In practice, multiple accumulative average is generally adopted to improve the SNR. M is the cumulative laser pulse number.

### 2.2. Optimization of Lidar Parameters

The goal of this paper is to develop a miniaturized lidar sensor; the effective detection range can reach 5 km in vertical detection and horizontal detection, the weight of the lidar sensor is limited to 15 kg in order to be installed on different platforms, such as scanning platform and vehicle for atmosphere detection. To achieve this goal, the parameters of the optical system are optimized first.

According to Equation (11), we take one parameter to be optimized as the independent variable, while the remaining parameters take the initialized value. Within the value range of the independent variable, the most effective detection distance of $Z_{max}$ corresponding to $SNR(\lambda ,Z_{max}$) = 3 is taken as the optimal value. The five parameters to be optimized are wavelength of laser, energy of laser pulse, diameter of telescope, FOV of telescope and bandwidth of filter; their values are shown in Table 1. Other parameters are determined according to design experience and actual use, such as laser beam divergence is 0.7 mrad and the repetition frequency is 5 kHz (depending on the inherent parameters of the laser). The transmittance of the beam expander of the transmitter unit can reach 0.9, the reflectance of other mirrors can reach 0.9 and the transmittance of the transmitter unit $T_{T}$ can reach 0.8. Usually, the transmittance of telescope is about 0.8, and the transmittance of narrow band filter is about 0.5, so $T_{R}$ is 0.4. For aerosol detection lidar, a spatial resolution of 7.5 m is sufficient, corresponding to a sampling time Δt 50 ns. M is 50,000. This is a balanced choice between SNR and temporal resolution. The aerosol extinction coefficient is obtained from Wu’s [23] statistical analysis. The sky spectral radiance [24] of different wavelength is 0.03 W/m^2^/Sr/nm @355 nm, 0.12 W/m^2^/Sr/nm @ 532 nm and 0.05 W/m^2^/Sr/nm @1064 nm. The usual quantum efficiency of detector is 30% (355 nm by PMT), 40% (532 nm by PMT) and 3% (1064 nm by APD) [25]

The distribution and variation of the atmosphere are complex, and the SNR ratio is an important index to judge the lidar detection performance. We simulate the SNR ratio of the lidar echo signal in the vertical detection case to optimize the parameters and verify the detection performance.

**Figure 2:**
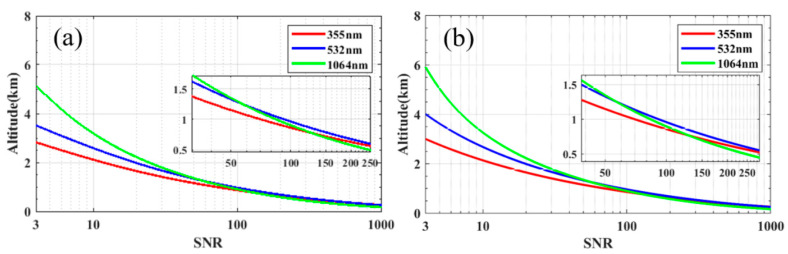
SNR curves for lidar wavelength of 355 nm (red), 532 nm (blue), 1064 nm (green) at daytime (**a**) and nighttime (**b**).

The wavelength of the laser is the first parameter to be determined. Figure 2 shows simulated curves of the SNR at three wavelengths (355 nm, 532 nm and 1064 nm), for FOV ω of 3 mrad, P_L_ of 10 μJ, A_R_ of 100 mm, θ of 1 nm and other parameters listed in Table 1, in daytime (Figure 2a) and nighttime (Figure 2b) conditions. The horizontal axis is the SNR and the vertical axis is the detection distance. From Figure 1 we find that Z_max_ becomes shorter as the wavelength reduces in both daytime and nighttime. For example, Z_max_ decreases from 5.1 km (1064 nm) to 2.8 km (355 nm) in daytime conditions, and Z_max_ decreases from 5.9 km (1064 nm) to 3.0 km (355 nm) in nighttime conditions. Longer wavelengths result in smaller extinction, and the signal is less attenuated by the atmosphere, so 1064 nm has a longer Z_max_ than 532 nm and 355 nm. However, shorter wavelength has better performance in the short range (below 1.25 km). This is because shorter wavelengths result in stronger backscattering, so the SNR is better. Therefore, for far distance detection (larger than 1.25 km), choose long wavelength 1064 nm, while for near distance detection (below 1.25 km), choose short wavelength 355 nm. In order to achieve a far detection distance, the preferred λ is 1064 nm.

**Figure 3:**
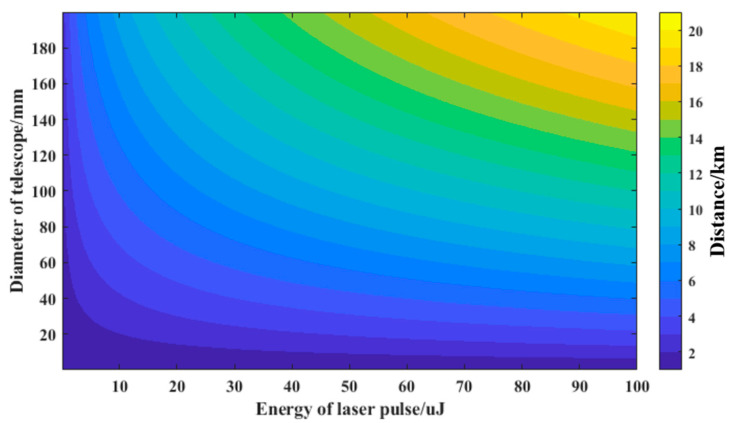
Maximum detection range map at different laser pulse energy and different Telescope diameter.

The next two parameters to be optimized are energy of laser pulse and diameter of telescope. The Z_max_ can be directly improved by increasing P_L_ and A_R_, but the volume, weight, power consumption and cost of lidar will also increase accordingly. We simulated Z_max_ under P_L_ and A_R_ in a certain range (P_L_ in 1–100 μJ, A_R_ in 1–200 mm), for λ of 1064 nm, FOV ω of 3 mrad, θ of 1 nm and other parameters listed in Table 1. In order to meet the requirement that the weight of the system is less than 15 kg, we evaluated A_R_ as 100 mm. To achieve the detection range of 5 km, P_L_ must be higher than 12 μJ according to Figure 3. Finally, P_L_ was determined as 15 μJ.

**Figure 4:**
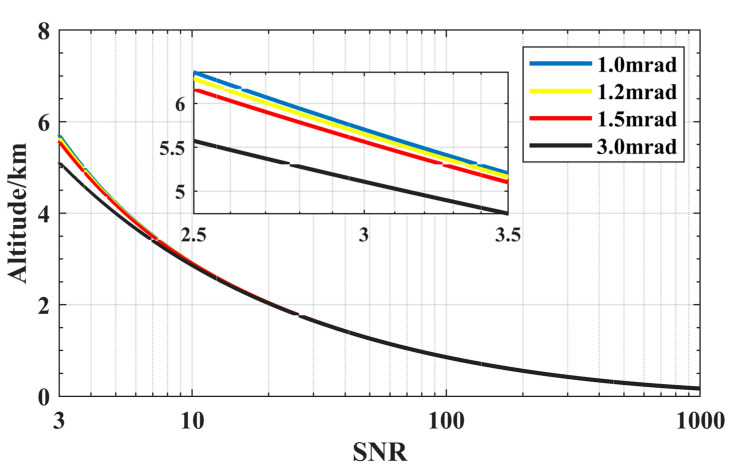
SNR curves of lidar corresponding to different FOV: 1.0 mrad (blue), 1.2 mrad (yellow), 1.5 mrad (red), 3.0 mrad (black).

Figure 4 shows simulated curves of the SNR at four FOV ω in daytime conditions, for λ of 1064 nm, P_L_ of 15 μJ, θ of 1 nm and A_R_ of 100 mm. As shown in Figure 4, Z_max_ increases as FOV ω decreases. The Z_max_ at different FOV ω are similar. The difference starts to increase after 4 km. For long-distance detection, a small FOV can minimize the receipt of sky spectral radiance. To ensure the full reception of signals, the FOV must be greater than the divergence angle of the laser. It is full of challenges in installation and adjustment of the laser and optical system with minimum FOV. Z_max_ under the 1.0 mrad FOV is only 0.2 km more than 1.5 mrad. Smaller FOV values do not improve Zmax much. The receiving field angle of 1.5 mrad is comprehensively considered. The value of FOV is selected as 1.5 mrad.

**Figure 5:**
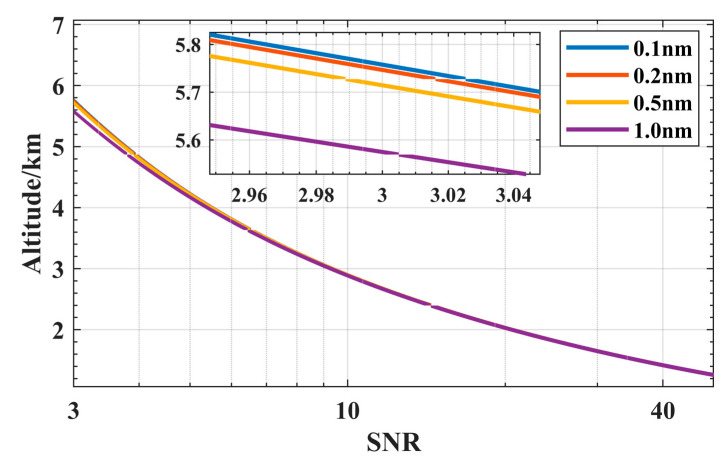
SNR curves of lidar corresponding to different filter bandwidth: 0.1 nm (blue), 0.2 nm (yellow), 0.5 nm (red), 1.0 nm (black).

The bandwidth of filter is the last parameter to be optimized. Simulated curves of the SNR at various bandwidths of filter θ, for λ of 1064 nm, P_L_ of 15 μJ, A_R_ of 100 mm and the FOV ω of 1.5 mrad, as shown in Figure 5. It is evident that as the filter bandwidth reduces, the detection range expands. However, the difference in Z_max_ with different bandwidth filters is not very large. The Z_max_ at 0.1 nm and 0.2 nm is only 50 m longer than Zmax at 0.5 nm. This is mostly due to the fact that the sky background radiation at 1064 nm wavelength is lower (in comparison to the visible band) [21]. A narrow filter can suppress the sky spectral radiance well, but it also reduces the transmission of the signal, which ultimately leads to a lower SNR. The Z_max_ at 0.5 nm is 150 m further than Zmax at 1.0 nm. In light of the aforementioned, we choose 0.5 nm filters that can satisfy the design requirements.

Finally, wavelength of laser, energy of pulse laser, diameter of telescope, FOV of telescope and bandwidth of filter were determined, and listed in the Table 2. The echo signal of lidar is simulated and the Z_max_ is 5.7 km in the daytime and 7.5 km at night.

**Figure 6:**
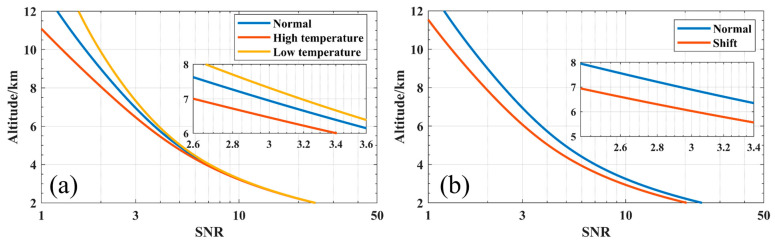
(**a**) Influence of Central wavelength of filter and (**b**) influence of dark count variation of APD on SNR at different temperature.

The temperature inside of the mIRLidar might vary from −40 °C to 50 °C in winter and summer when it runs outdoors. The hard-coated filters from Allxua typically exhibit a temperature-dependent wavelength shift in the operating temperature range of 2 pm/°C to 5 pm/°C [26,27]. The central wavelength of the filter may move by 0.2 nm in either direction due to significant temperature fluctuations. We simulated the effect of wavelength shift on the mIRLidar’s Z_max_, and Figure 6a shows that Z_max_ is lowered by about 1.0 km. This has a significant impact on the mIRLidar’s capacity to detect atmosphere. Therefore, the temperature and humidity should be steadily controlled when using narrow band filters [5]. The gain, dark current or dark count of APD detector are all significantly influenced by temperature. At dark count 300 counts/s, the single photon detector’s maximum change rate can be as high as 20% [28], and this has an impact on Z_max_ of about 1.0 km. In the meantime, temperature change will result in gain instability and error in signals of detector, causing the profile of atmospheric to be incorrect.

Therefore, it is necessary to ensure the stability of the filter and detector operating temperature to ensure the stable operation of the mIRLidar.

## 3. Development of mIRLidar System

In the previous section, we selected the best parameters through simulation calculation, as listed in Table 2. On this basis, we developed the hardware system. We effectively reduced the volume of the lidar through reasonable device stacking, while improving the heat dissipation of the internal components, optimized the structural design to reduce the weight of the lidar, and also designed a temperature control system to ensure the stable operation of the core components.

**Figure 7:**
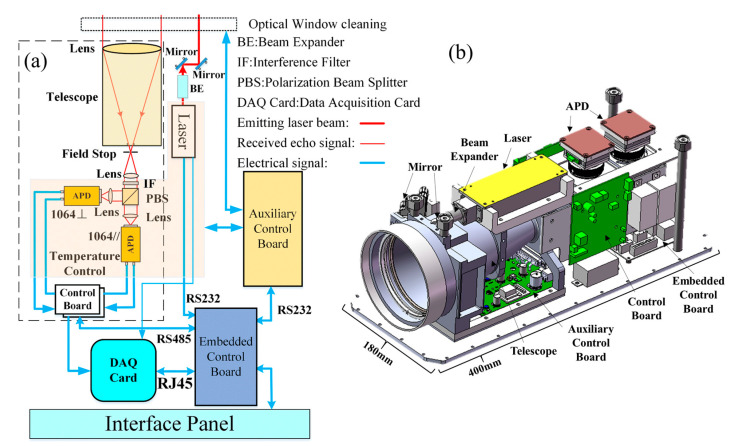
mIRLidar system: (**a**) schematic diagram and (**b**) internal structure diagram.

The structure of mIRLidar sensor is shown in Figure 7a, which mainly includes laser emission unit, signal receiving unit, data acquisition unit and auxiliary control unit. 

The model of mIRLidar is shown in Figure 7b. The transceiver system adopts an off-axis design. The receiving system uses an aspherical mirror with diameter 100 mm and focal length 200 mm to receive backscattered signals. The telescope tube adopts a tapered design and is processed with LY12 aluminum, which can minimize the size and weight. At the same time, the rough stripes on the inner wall can restrain some stray light. The telescope is also used as the reference of the optical mechanical part, The laser is installed on the telescope, and the subsequent optical path unit is connected to the rear end of the telescope, which can effectively ensure the stability of the optical path. In the laser emission unit, Liu [29] has provided us with a high performance and high stability laser. The laser center wavelength is 1064.2 nm. A 10-times beam expander is used to shape the beam. After shaping, the divergence angle is 0.7 mrad, and the single pulse energy is 15 μJ. The repetitive frequency can reach 10 kHz at most, and the overall dimension is 50 mm × 40 mm × 28 mm (corresponding to length, height and width, respectively). It can work stably under −50~70 °C without additional temperature control. The embedded controller controls the start and stop of the laser through the serial port. The auxiliary control panel is used for monitoring the temperature and humidity inside mIRLidar, controlling the temperature of the filter and cleaning the optical window, and is installed on the aluminum base plate under the telescope. The embedded control board (ECB) is used to replace the traditional industrial personal computer (IPC), which greatly reduces the volume, weight and power consumption of the system. ECB is mainly used for the configuration of device parameters, the collection and storage of signals, and is installed on the aluminum bottom plate under the rear optical path unit. The aluminum bottom plate can also heat the circuit board. The two APD detectors are installed on the corresponding channel of the rear optical path unit and driven by a control board respectively, which is fixed on both sides of the rear optical path unit. The shell of mIRLidar is made of lightweight and high-strength nylon. The shell is installed with high-speed acquisition card, power supply and network interface. The upper cover plate of the lidar is covered with heat sink for cooling the temperature control device of the laser and APD detector. Table 3 shows the main parameters of the mIRLidar.

**Figure 8:**
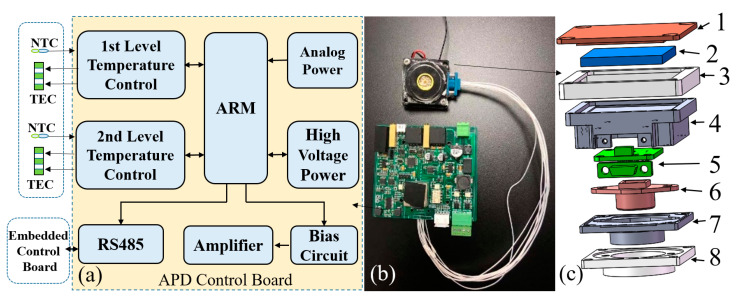
(**a**) Schematic diagram of APD control unit, (**b**) photo of APD detector and (**c**) internal structure diagram of APD: (1) Cu-heat sinks; (2) TEC; (3) nylon cover; (4) metal base; (5) PCB; (6) APD; (7) metal base; (8) nylon base.

The detector and filter are sensitive to temperature, and the conventional detector and filter can only work at room temperature. We designed a special structure and control system based on TEC (Thermo Electric Cooler) to ensure the temperature stability. We designed the APD control board based on ARM (Advanced RISC Machines, a microprocessor model) as the controller, including analog power supply, high-voltage power supply, two-stage temperature control circuit and signal processing circuit. The first stage temperature control circuit is responsible for the accurate temperature control of the target surface of the APD detector, and the second stage temperature control circuit is responsible for controlling the ambient temperature of the APD enclosure at room temperature. Figure 8c shows the structure of the APD detector. The APD is packaged with aluminum structures (4) and (7) and injected with thermal conductive adhesive at the same time. The heat of the APD can be well exported. The electrical pin is led out through the PCB (Printed Circuit Board) (5). When the temperature in the equipment deviates from the room temperature, the semiconductor refrigeration chip in the APD is driven by the primary temperature control circuit to control the temperature of the target surface. When the temperature in the equipment exceeds the threshold, the secondary temperature control is started. The ambient temperature of the APD shell is controlled within the room temperature value through the semiconductor refrigeration chip, which reduces the pressure of the primary temperature control, and ensures that the APD target surface can work stably at a lower temperature. The upper cover plate is used for the heat dissipation of the TEC hot surface.

**Figure 9:**
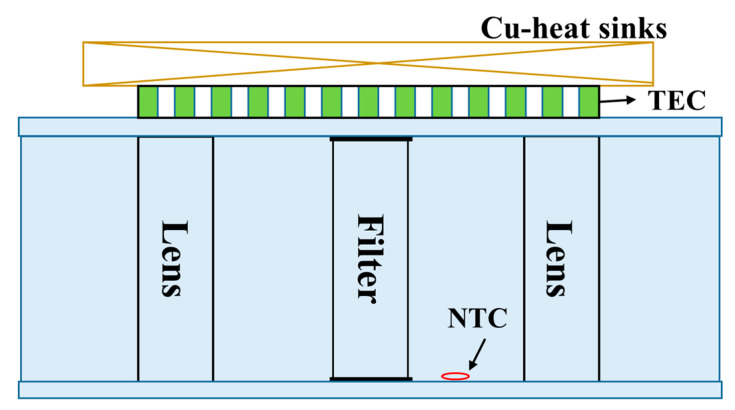
Schematic diagram of filter temperature control.

The filter is installed at the connection between the optical path unit and the telescope to filter out the solar radiation, in order to reduce the interference to the mIRLidar signal. TEC is installed outside the connection. The hot side is connected with a copper plate for heat dissipation. The two lenses installed at the front and back of the filter form a closed cavity to protect the filter from being polluted by condensed water vapor. A temperature sensor is installed in the closed cavity, with the structure shown in Figure 9. By changing the current direction through TEC, TEC can realize the cooling or heating of the filter enclosure. A closed loop negative feedback control circuit is designed to automatically control the temperature of the filter closure box. The temperature is controlled at 25 ± 0.5 °C to ensure the stable central wavelength and transmissivity of the filter.

We conducted a high–low temperature test on the whole mIRLidar to verify the performance of the designed temperature control system and the temperature adaptability of mIRLidar. We put the mIRLidar in the high–low temperature chamber; the temperature of the detector was stably controlled at −5 °C, and the temperature at the filter was stably controlled at 25 °C.

**Figure 10:**
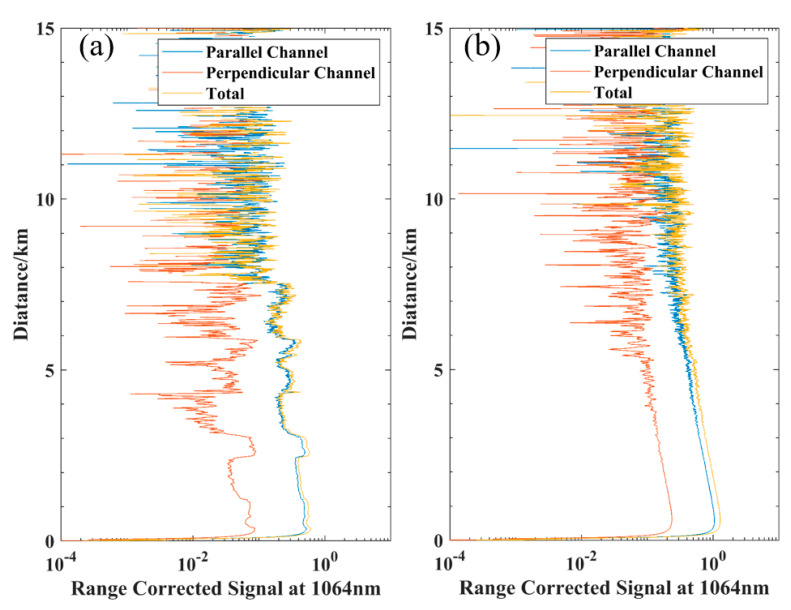
Detection distance test, (**a**) vertical detection and (**b**) horizontal detection.

After completing the development, we tested the detection performance of the mIRLidar in Hefei (117.128496° N, 31.828237° E) on 13 March 2022, at 0:00–4:00 (LT). The maximum detection distance in vertical and horizontal direction was tested, respectively, under the weather conditions of AQI (Air Quality Index) about 95, PM_2.5_ about 55 μg/m^3^, PM_10_ about 100 μg/m^3^ and wind force was level 2 (Data from China National Environmental Monitoring Centre, http://www.cnemc.cn/en/ accessed on 30 December 2022), and the raw data were collected at resolutions of 20 s and 7.5 m. Figure 10a (at 0:00, LT) shows the range corrected signal (RCS) profiles in the vertical detection. The RCS profile shows that the effective detection height in the vertical detection is greater than 6.0 km, where there is a thin cloud or aerosol layer above 5 km. Figure 10b (at 3:35, LT) shows the RCS profiles in the horizontal mode. The RCS profile shows that the effective detection distance in the horizontal detection can reach 8 km. The above tests suggest that the effective detection distance of the lidar is consistent with our previous parameter optimization.

## 4. Observations

**Figure 11:**
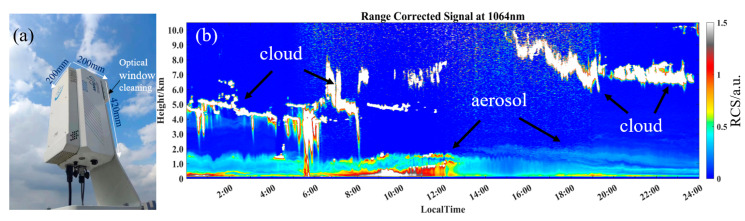
(**a**) Photo of mIRLidar system and (**b**) RCS profiles in vertical mode on 14 March 2022, in Hefei (UTC+8).

After completing the mIRLidar performance test, we used it to perform some atmospheric detection experiments, and also verified the stability of the mIRLidar. Different detection modes and different scene applications can be realized through simple external connection devices, benefiting from the excellent detection performance, compact size and low power consumption of the mIRLidar. By installing a bracket, the mIRLidar is installed vertically upward, as shown in Figure 11a. It can perform unattended work, only by providing the mIRLidar with a stable external power and network. mIRLidar will automatically start detection when the power is started, the data will be synchronized to the server through the network.

We conducted a continuous detection experiment with 7.5 m vertical and 20 s temporal resolutions in Hefei (117.128496° N, 31.828237° E) on 14 March 2022, and the weather was cloudy. From Figure 11b, it can be seen that the atmospheric troposphere presents an obvious hierarchical structure, and the distribution and change in aerosols in the boundary layer are accurately detected, from which we can see the change in the boundary layer height in a day, which is consistent with the change rule of the boundary layer in Hefei in spring [30]. At the height of about 4 km, an aerosol layer, a multi-layer cloud structure and a cloud above 10 km were also detected. The mIRLidar can be used to detect the continuous vertical structure of the atmosphere, the height of the boundary layer and the change process of aerosols, which is of great significance to the study of local climate change, and can provide important data for specifying air pollution prevention and control strategies [30].

**Figure 12:**
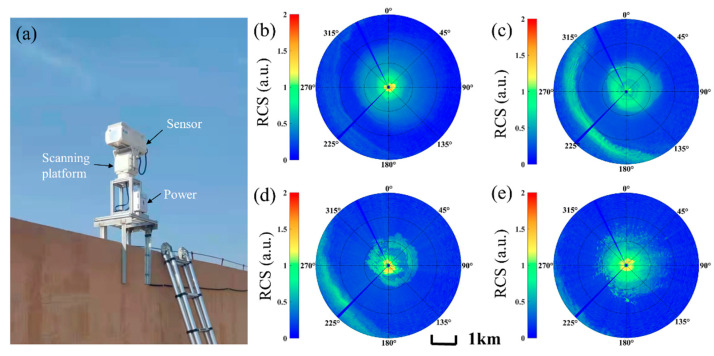
(**a**) Photo of mIRLidar system and RCS maps (3 km radius) in scan mode at different times on 17 March 2022, in Zibo (UTC+8): (**b**) 06:00~06:30, (**c**) 06:30~07:00, (**d**) 07:00~07:30 and (**e**) 07:30~08:00.

To detect the distribution characteristics of atmospheric aerosols in three-dimensional space, we installed mIRLidar with a weight of 13.5 kg on the scanning platform, as shown in Figure 12a. It can rotate 360° horizontally and ±90° vertically. We conducted continuous scanning detection experiments in an industrial park in Zibo (37.048553° N, 117.874941° E), Shandong, during 17 March 2022. The installation point is located on the roof of the building at a height of about 15 m. The 0° angle corresponds to the north direction, 180° is the south direction, and the step angle is 2°. There are 180 datapoints with 7.5 m vertical and 10 s temporal resolutions. It takes 30 min to scan one circle. From Figure 12b, it can be seen that there is a long aerosol tape about 2~3 km in the west of the detection point, in the period from 06:00 to 06:30 (LT), with a low concentration. The concentration increases in the following half an hour. The whole aerosol tape is gradually transmitted and diffused to the southwest under the action of northeast wind in the period from 07:00 to 07:30 (LT), and diffuses outside the detection range in the time period from 07:30 to 08:00 (LT). The scanning detection of lidar can achieve a wide range of atmospheric aerosol, mIRLidar can be used for the identification of atmospheric pollution by combining with data of in-situ monitoring stations, and can provide a powerful monitoring for pollution emission control.

**Figure 13:**
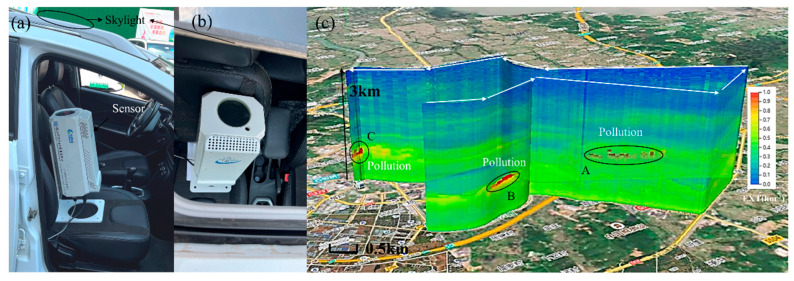
(**a**,**b**) Photo of mIRLidar system, and (**c**) extinction maps in Navigation detection mode on 25 April 2022, in Quanzhou (UTC+8).

We installed mIRLidar on the car with a skylight for navigation detection to explore the vertical structure of the atmosphere at different positions in the area, as shown in Figure 13a. Due to the small size, low power consumption and low requirements for the navigation car, it overcomes the disadvantage of the mobile lidar developed by Xie [20] which is unable to observe while moving. The mIRLidar, due to its low power consumption and relatively long operating time, is able to detect while moving. mIRLidar can detect atmospheric signals over the moving path rather than mobile lidar only can change from one place to another to detect. On 15 April 2022, we conducted a navigation detection experiment in Quanzhou (118.678887° N, 24.881198° E), Fujian Province. The mIRLidar is powered by a mobile inverter power supply, the approximate power of mIRLidar is about 50 W. The speed was about 30 km/h, and the whole detection lasted about half an hour, with a distance of about 12.5 km. mIRLidar collected a profile every 10 s. After the mIRLidar was started, the navigation car drove at a constant speed on the flat road. An industrial controller recorded each detection datapoint and corresponding location information, and displayed the data through GIS (Geographic Information System). The weather conditions in Quanzhou were AQI about 22, PM_2.5_ about 15 μg/m^3^, PM_10_ about 27 μg/m^3^ (Data from China National Environmental Monitoring Centre, http://www.cnemc.cn/en/ accessed on 30 December 2022) and there were no clouds in the sky. We used the Fernald method [20] to invert the extinction coefficients of atmospheric aerosols. From Figure 13b, can be seen clearly that the vertical structure distribution of the atmosphere on the route was different at different locations. Three pollution spots were detected during the navigation. Among them, there are many scattered pollution spots at 0.5 km near point A, and one pollution spot at 0.8 km near point B and C, respectively. Since these three pollution spots are discontinuous with the ground, it is analyzed that they may be transmitted from other nearby locations.

## 5. Conclusions and Outlook

We established a lidar simulation model based on the lidar equation and atmospheric model, and completed the parameter optimization and performance simulation before the development of mIRLidar. This design approach can provide a reference for the development of other Lidar systems. The mIRLidar system with a 1064 nm laser (the pulse laser energy 15 μJ, the repetition frequency 5 kHz), a 100 mm aperture telescope (the FOV 1.5 mrad), 0.5 nm bandwidth of filter and specially designed APD. The filters and detectors were designed for additional temperature control. The mIRLidar system has a volume of 200 mm × 200 mm × 420 mm and weighs about 13.5 kg. Its effective detection range can reach 5 km in most weather conditions and is consistent with our previous parameter optimization. Horizontal and scanning atmospheric measurements have been carried out to validate its performance and stability. It can effectively detect the vertical distribution of aerosols in the boundary layer and the high cloud structure. The scanning and navigation detection experiments show that mIRLidar can be used to detect the distribution and transmission of atmospheric pollution and the vertical distribution of regional pollution.

The mIRLidar can also be used for airport visibility detection and real-time monitoring of expressway cloud fog [31]. In the future, three-dimensional detection of the boundary layer atmosphere can be achieved through multiple platforms and multi-dimensional detection such as ground-based vertical detection, ground-based scanning detection and unmanned aerial detection, providing detection data for meteorological research and environmental monitoring.

## Figures and Tables

**Table 1:** Parameters to be optimized and Optimal values of mIRLidar.

Parameters	Index	Values
λ	Wavelength of laser	355 nm, 532 nm, 1064 nm
η	Quantum efficiency of detector	30% (PMT @355 nm) 40% (PMT @532 nm) 3% (APD @1064 nm)
$P_{B}$	Sky spectral radiance	0.03 W/m^2^/Sr/nm (@355 nm) 0.12 W/m^2^/Sr/nm (@532 nm) 0.05 W/m^2^/Sr/nm (@1064 nm)
$P_{L}$	The energy of laser pulse	1~100 μJ
ω	FOV of telescope	1.0 mrad, 1.2 mrad,1.5 mrad,3.0 mrad
$A_{R}$	Diameter of telescope	1~200 mm
θ	Bandwidth of filter	0.1 nm, 0.3 nm, 0.5 nm, 1.0 nm
$\lambda_{0}$	The central of the filter(affected by temperature)	1064 nm
$C_{D}$	Dark count(affected by temperature)	300

**Table 2:** Optimized optical parameters of mIRLidar.

Parameters	Value
Wavelength of laser	1064 nm
Energy of pulse laser	15 μJ
Diameter of telescope	100 mm
FOV	1.5 mrad
Bandwidth of Filter	0.5 nm

**Table 3:** Main parameters of mIRLidar.

Transmitter	
Laser wavelength	1064.2 nm
Laser beam Divergence	0.7 mrad
Energy of pulse laser	15 μJ
Pulse repetition	5 kHz
Pulse width	3 ns
Beam size	2.5 mm
**Receiver**	
Diameter of telescope	100 mm
FOV	1.5 mrad
Bandwidth of filter	0.5 nm
Detector	APD
Acquisition mode	AD/16 bit
Sampling rate	20 MHz
**System**	
Size	200 × 200 × 420 mm
Weight	~13.5 kg
Power	<100 W
Operating temperature	−40~50 °C

## Data Availability

Data underlying the results presented in this paper are available by contacting the first author or the corresponding author.
